# Preparation and Immunogenicity Evaluation of a Ferritin-Based GnRH Nanoparticle Vaccine

**DOI:** 10.3390/vaccines13080781

**Published:** 2025-07-23

**Authors:** Ying Xu, Weihao Zhao, Yuhan Zhu, Bo Sun, Congmei Wu, Yuhe Yin

**Affiliations:** 1 College of Chemistry and Life Sciences, Changchun University of Technology, Changchun 130012, China; 2202308102@stu.ccut.edu.cn (Y.X.); 1202308010@stu.ccut.edu.cn (W.Z.); 2202308109@stu.ccut.edu.cn (Y.Z.); wucongmei@ccut.edu.cn (C.W.); 2 School of Life Sciences, Jilin University, Changchun 130012, China; bo_sun@jlu.edu.cn

**Keywords:** GnRH, immune castration, ferritin, nanoparticle vaccines, Spy Catcher-Spy Tag

## Abstract

**Objectives**: Research on the immunocastration vaccine is of great significance for animal management. In this study, the gonadotropin-releasing hormone (GnRH) ferritin nanoparticle vaccine was constructed using Spy Catcher-Spy Tag (SC-ST) as a delivery system; **Methods**: The Spy Catcher was constructed to fuse with the expression vector pET-30a-SF of ferritin nanoparticles. Two polypeptides, STG1: Spy Tag-GnRH I-PADRE and STG2: Spy Tag-GnRH I-GnRH II, coupled to SF in vitro to form two nanoparticles, were designed and synthesized to detect castration effects in mice. We mixed them with the adjuvant MONTANIDE ISA 206 VG to explore the adjuvant’s effect on immunogenicity; **Results**: All immunized groups produced anti-GnRH specific antibodies after the second immunization, which was significantly higher in the immunized group and the combined adjuvant group than in the control group, and the immune response could still be detected at the 12th week. The concentrations of testosterone, follicle-stimulating hormone, and luteinizing hormone in serum were significantly decreased. The number of sperm in the epididymis of mice in each immune group was significantly reduced, and the rate of sperm deformity was high; **Conclusions**: The two ferritin-based GnRH nanoparticles developed in this study can significantly cause testicular atrophy, decreased gonadal hormone concentration, decreased sperm count, and increased deformity rate in male mice. These findings provide experimental evidence supporting their potential application in animal immunocastration.

## 1. Introduction

Castration refers to the removal or inhibition of the function of the reproductive glands (testes or ovaries) by surgical or chemical methods, thereby blocking the secretion and reproductive capacity of sex hormones, and it is of great significance in pet management and animal husbandry. Immunocastration refers to the technique of inhibiting the development of sexual organs with immunologic methods to achieve the purpose of castration [1,2]. This technique blocks the reproductive hormone-regulated pathway, mainly by inducing antibodies directed against key antigens during reproduction, through specific reactions between antigens and antibodies, thus achieving the goal of controlling fertility [3,4]. Immunocastration has the advantages of safety and convenience, mild effect, less stress in animals, and reversibility.

Gonadotropin-releasing hormone (GnRH) is a decapeptide hormone secreted by the mammalian hypothalamus that regulates the reproductive system. It regulates the reproductive function and behavior of animals through the hypothalamic–pituitary–gonadal axis (HPG axis). It is a central initiating and regulatory hormone in animal reproductive physiology [5,6]. Studies have confirmed that GnRH antibodies can specifically neutralize endogenous GnRH in organisms, leading to partial or complete loss of GnRH biological activity in vivo, disrupting the body’s inherent HPG axis balance, reducing gonadal hormone levels, and achieving immunocastration [7,8]. However, GnRH has low immunogenicity, which was enhanced in vaccine studies by altering the structure of GnRH or by coupling GnRH with carrier proteins. Faruck MO et al. [9] immunized both mice and pigs with GnRH peptide conjugated to PMA polymer and T helper epitope, induced high titer IgG antibody levels in animal models, reduced pregnancy rates by more than 80% in mice, and maintained antibodies for more than 6 months in pig models, without toxicities or immune tolerance.

Ferritin nanoparticles (NPs) exhibit octahedral symmetry, with subunit assemblies forming structures that have an 8-nm inner diameter and a 12-nm outer diameter [10,11,12]. Ferritin NPs are widely used in vaccine development as they act as both delivery vehicles and adjuvants, binding tightly to antigens and functioning synergistically [13]; compared with soluble antigens, ferritin-based self-assembled nano-platforms can significantly increase antibody titers to form a robust and long-lasting immune response [10,14,15,16]. Recent advances in ferritin engineering, including surface modifications and subunit reprogramming, further expand their applicability in vaccine design [17,18,19].

Spy Tag/Spy Catcher is a protein-coupled method produced by the CnaB2 domain isolated from the Streptococcus pyogenes fibronectin FbaB [20]. Spy Catcher is a 113-amino acid protein containing lysine (Lys31), the key reactive site; and Spy Tag is a 13-amino acid short peptide with a serine (Asp117) reactive site. After mixing, Spy Tag and Spy Catcher bind and spontaneously undergo an amidation reaction promoted by the Spy-Catcher residue glutamate (Glu77), forming an intermolecular isopeptide bond [21]. The key to the modular nanoparticle design is the embedding of Spy Catcher into the nanoparticle surface, allowing the Spy Tag to chemically connect with the target protein, which is able to attach to the nanoparticle surface when the Spy Tag-linked protein comes into contact with the Spy Catcher-modified nanoparticle. Several studies have confirmed the effectiveness of vaccine vectors based on Spy Tag/Spy Catcher and ferritin [22,23]. Notably, Chang et al. [24] fused a Spy Tag short peptide to the C-terminus of ferritin and a Spy Catcher protein to the N-terminus of the Porcine Reproductive and Respiratory Syndrome Virus cell epitope. These were highly coupled via the Spy Tag/Spy Catcher isopeptide bond with an efficiency exceeding 99%. The resulting vaccine was significantly superior to conventional vaccines in both mouse and piglet models.

Despite these advancements, there are no relevant reports of the construction of castration vaccines using a nanocarrier system based on Spy Tag/Spy Catcher with ferritin. Herein, we present a novel GnRH vaccine platform leveraging this system: Spy Catcher was fused to ferritin NPs, while tandem GnRH I/GnRH II epitopes and PADRE T-helper motifs were conjugated via Spy Tag. Through comprehensive murine immunization studies, we evaluated the vaccine’s capacity to suppress gonadal hormones, induce testicular atrophy, and impair spermatogenesis, thereby establishing a foundation for next-generation immunocastration strategies.

## 2. Materials and Methods

### 2.1. Plasmids, Strains, Polypeptides, and Experimental Animals

The pET-30a-SF plasmid was synthesized by Nanjing GenScript Biotech Co., Ltd. (Nanjing, China). *E. coli BL21 (DE3)* competent cells were purchased from Beijing TransGen Biotech Co., Ltd. (Beijing, China). Polypeptides STG1 and STG2 were synthesized by GL Biochem Co., Ltd. (Shanghai, China). Seventy-seven male BALB/c mice (4-week-old, specific pathogen-free) were obtained from Changchun Yisi Laboratory Animal Technology Co., Ltd. (Changchun, China). The animals were housed under controlled conditions provided by Changchun Xinuo Co., Ltd. (Nanjing, China).: barrier-system facility with room temperature maintained at 19–23 °C, relative humidity 40–65%, alternating light 12 h bright and 12 h dark, and independent ventilation cages (IVC). All cages and pads were used after autoclaving, and mice had ad libitum access to food and water.

All animal experiments were approved by the Animal Ethics Committee of Changchun Long Sheng Experimental Animal Technology Co., Ltd. (Changchun, China). (CCLSLL-2024110703), and the experimental operations were subject to the “Guidelines for the Welfare and Ethics of Experimental Animals in China”.

### 2.2. Construction and Expression of the Recombinant Plasmid pET-30a-SF

Ferritin was linked to Spy Catcher by ligand peptide (GGGGS), with histidine (6×His) labeling added at the end. Two enzymatic cleavage sites, Bam H I and Hind III, were selected, and the sequences were cloned into the pET30a vector (synthesized by Nanjing Kingsley Biotechnology Co., Ltd. (Nanjing, China). The plasmid pET-30a-SF (SF: SpyCatcher-ferritin) was constructed, and sequencing identification was performed. Recombinant plasmid pET-30a-SF was transfected into *E.coli BL21(DE3)* competent cells, cultured at 37 °C, 220 rpm to an OD600 value of 0.6–0.8, and expressed for 14 h with 0.25 mM IPTG added at 25 °C, 220 rpm.

### 2.3. Purification and Identification of Recombinant SF Proteins

The supernatants of the bacteria after induction of expression were collected, and the final concentrations of 20%, 25%, 30%, 35%, 40%, and 45% were selected for saturation ammonium sulfate purification to determine the optimal saturation ammonium sulfate concentration. After the filtration of supernatants after the second centrifugation, purification was performed. Purification was performed by hydrophobic chromatography Phenyl-30L columns, ion-exchange chromatography NanoGel-50Q, buffer A balanced 5 column volumes, and hydrophobic chromatography Diamond Butyl. Peak samples were collected at each stage and SDS-PAGE was performed to detect the target protein.

### 2.4. Construction of the Polypeptide STG1, STG2

NCBI published the amino acid sequences of Spy Tag (PDB: 4MLI_B), GnRH I (GenBank: AAF78457.1), GnRH II (GenBank: AAD48032.1), and the artificially designed T helper cell (Th) epitope PADRE (AKFVAAWTLKAAA), constructing STG1: Spy Tag-GnRH I-PADRE, and STG2: Spy Tag-GnRH I-GnRH II, respectively, synthesized by Jill Biochemical Co., Ltd. (Shanghai, China).

### 2.5. Preparation and Characterization of SF-STG1 and SF-STG2 Nanoparticles

SF was mixed with STG1, and SF was mixed with STG2, respectively, at a molar ratio of 1:8 and incubated overnight at 4 °C. The optimal coupling efficiency of the proteins mixed overnight was evaluated using 15% SDS-PAGE. Samples were placed on carbon-coated copper grids for adsorption over 60 s. After removing excess samples with PBS, the grids were stained with 2% (*w*/*v*) phosphotungstic acid (PTA) for 60 s. The negatively stained samples were dried, and 10 µL of each sample was dropped onto the coated copper grids, followed by incubation for 5 min. The grids were rinsed three times with distilled water, then stained with 5 µL of uranyl acetate for 3–5 min. After air-drying, the samples were observed under a transmission electron microscope (TEM). Additionally, the particle size distribution of the nanoparticles was assessed by dynamic light scattering (DLS) at 25 °C.

### 2.6. Preparation of Immunogenic Agents and the Immunization of Mice

Four-week-old Balb/c mice were selected and placed in a clean level animal room in 12-h light and 12-h dark cycles with free feed and water. Forty-two mice were randomly divided into six groups, each with a first immunization at 0 w, a booster at 2 w, and 4 w; serum was harvested every 2 weeks, mice were sacrificed at 12 w, and the immunization was by subcutaneous injection. Protein immunization groups were set up: STG1, SF-STG1, STG2, SF-STG2, PBS control group, and SF control group, with mouse immunorecombinant protein doses of 20 μg diluted in PBS to 100 μL.

To explore the effect of adjuvants on immunogenicity, 4-week-old Balb/c mice were placed in a greenhouse, illuminated for 12 h and dark for 12 h, freely fed with feed and water. Thirty-five mice were randomly divided into 5 groups, each with the first immunization at 0 w, booster immunization at 2 w and 4 w, and serum sampling every 2 weeks. Mice were sacrificed at week 12; the immunization was by subcutaneous injection. Protein immunization groups were set up: SF-STG1, SF-STG1+ISA 206, SF-STG2, SF-STG2+ISA 206, and PBS control groups with mouse immunorecombinant protein doses of 20 μg diluted in PBS to 100 μL. Among them, ISA 206 adjuvants were all mixed with nanoparticles in a 1:1 ratio to prepare the vaccine.

The experimental animals and experimental operations involved in the paper have been approved by the Ethics Committee and strictly followed the rules and regulations of animal experiments. After the experiment, all experimental animals were treated humanely (amniotic fluid) to minimize their pain and harm, in accordance with animal ethics-related norms.

### 2.7. Sample Collection

Peripheral blood samples were collected from the tail vein of each mouse every 2 weeks, coagulated in a centrifuge tube for 1 h, and centrifuged at 3000 rpm for 30 min, and serum was collected and stored at −80 °C. Mice were sacrificed and testicles were removed at 12 w postimmunization, measured in length, width, and weighed, and stored in 4% paraformaldehyde solution. Sperm was collected from the tail of the epididymis and stored in BWW sperm culture medium (Beijing Reagan Biotechnology Co. CM0052, Beijing, China).

### 2.8. ELISA Determination of the Specific GnRH Antibody

Flat-bottom 96-well plates were coated with 1 μg of GnRH polypeptide in coating buffer (100 μL/well, pH 7.4) and incubated overnight at 4 °C. Plates were washed 5 times with 0.05% (*v*/*v*) Tween 20 for 1 min each time, and then blocked with 2% bovine serum albumin (200 μL/well) for 1 h at 37 °C. Then, the blocking buffer was discarded, washed 5 times with PBS buffer containing 0.05% (*v*/*v*) Tween 20, diluted serum (200 μL/well; 1:50) was added to samples in duplicate, and it was blocked for 1 h at 37 °C. After washing 5 times with PBS buffer containing 0.05% (*v*/*v*) Tween 20, HRP-conjugated goat anti-mouse IgG antibody 1:5000 (Beyotime, Nanjing, China) was added, plates were incubated for 1 h at 37 °C, plates were washed 5 times, and 100 μL of TMB substrate was added to each well for a light-avoidant reaction for 10 min. The reaction was stopped by adding 50 μL 2 M sulfuric acid solution to each well. The absorbance of each well was measured with a microplate reader at a wavelength of 450 nm, and the results were expressed as the optical density (OD) value.

### 2.9. Determination of Serum Concentrations of Testosterone (T), Follicle-Stimulating Hormone (FSH), and Luteinizing Hormone (LH)

The collected serum was assayed for T, FSH, and LH concentrations by the ELISA method using the Joint Immunosorbent Assay (ELISA) kit, and the experimental steps were performed as indicated by the kit.

### 2.10. Hematoxylin- and Eosin-Stained Section of a Testicular Tissue

After mice were sacrificed, the testicles were dissected for morphological measurements, blood vessels, fat, and connective tissue were removed, and testicular weights were recorded for each mouse. Testicular tissue was fixed in 4% paraformaldehyde solution for 24 h. After fixation, the tissues were washed and embedded in a single paraffin block for histological evaluation. Sections (5 μm) were fixed on slides, heated, and dried at 37 °C for 24–36 h. Slides were deparaffinized, washed again by gradient ethanol bath, and stained with hematoxylin and eosin stain. Slides were dried for 4–8 h in a 37 °C incubator and mounted using cover slips. Slides were examined under a microscope with magnification of 40, 100, 200, and 400 for morphological observation.

### 2.11. Sperm Collection and Quality Analysis

While the mouse testis was removed, the epididymis was isolated to collect semen. The tail of the epididymis was trimmed to remove fat and connective tissue, washed in PBS, and placed in 500 μL of BWW medium. Each tail was cut into 4–6 sections and semen was released into the medium by incubation at 37 °C and 5% CO_2_ for 1 h. After incubation, tissues were removed and the suspension was gently mixed by pipette for sperm morphology and quality analysis.

Spermatozoa counts were measured using Neubauer’s blood cell counter, and sperm motility was assessed by microscopy (20 μm). Sperm morphology was assessed using air-dried Giemsa staining and eosin-black rosin staining to determine the proportion of normal and abnormal sperm. The sperm quality was assessed by the number of sperm and deformity rate in 200–500 sperm in each mouse semen sample.

### 2.12. Statistical Analysis

Intergroup differences were analyzed using Graphpad Prism 8.0 software, with *t*-tests comparing two groups and one-factor ANOVA comparing multiple groups. The data for intergroup differences were expressed as mean ± SD (*, 0.01 < *p* < 0.05; **, *p* < 0.01, ***, *p* < 0.001; significant difference ns, no significant difference).

## 3. Results

### 3.1. Recombinant Plasmid Identification

**Figure 1:**
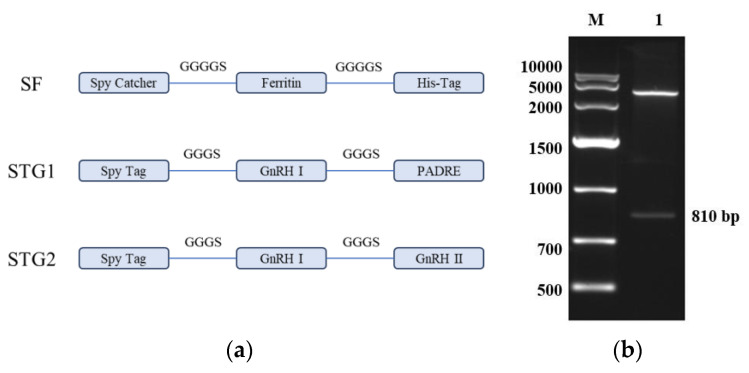
(**a**) Schematic design of the ferritin nanoparticle-based vaccine platform. (**b**) Restriction enzyme digestion analysis of the recombinant plasmid pET-30a-SF M: DNA Marker DL10,000; 1: The recombinant plasmid pET-30a-SF was subjected to digestion with Nde I and Hind III.

As shown in Figure 1a, the SpyCatcher sequence was inserted into the N-terminus of the Ferritin protein sequence via a flexible GGGGS linker, with an added His-Tag. After codon optimization for Escherichia coli, the sequence was ligated into the pET30a(+) vector to construct the recombinant plasmid pET30a-SF. The SpyTag was fused to GnRH I and PADRE amino acid sequences using GGGS linkers, synthesizing the polypeptide STG1. Similarly, SpyTag was connected to GnRH I and GnRH II amino acid sequences via GGGS linkers, yielding the polypeptide STG2.

As shown in Figure 1b, the results of agarose gel electrophoresis showed that the enzymatic cleavage products presented two specific bands in the electrophoretic profiles, which were molecularly consistent with the theoretical predictive values of the empty vector pET-30a and the Spy Catcher-Ferritin fusion genes, respectively. The accuracy of the construction of the recombinant plasmid pET-30a-SF was further verified by a comparative analysis of nucleic acid fragment mobility.

### 3.2. Recombinant Protein Identification, Purification, and Characterization

**Figure 2:**
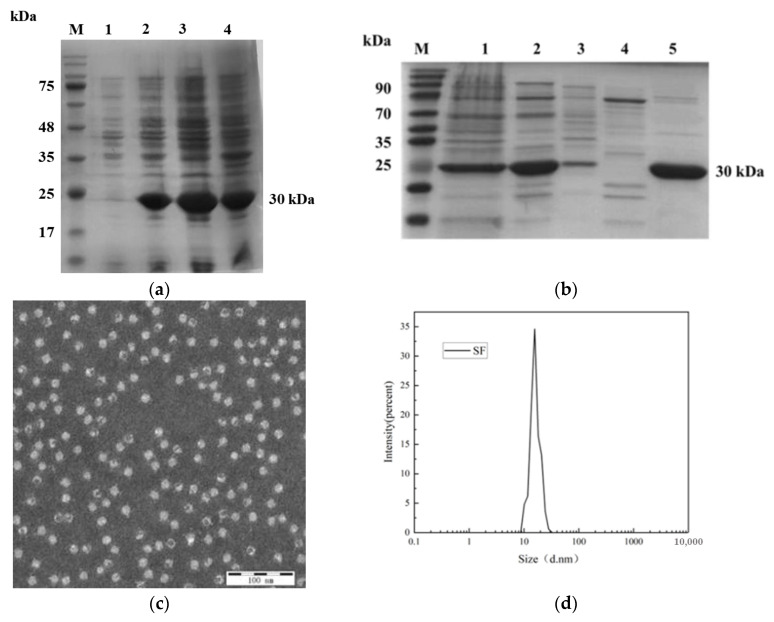
(**a**) Analysis of recombinant protein expression. M: Protein marker; Lane 1: pET-30a-SF before induction; Lane 2: pET-30a-SF after induction; Lane 3: Supernatant of pET-30a-SF after sonication; Lane 4: Pellet of pET-30a-SF after sonication. (**b**) Purification profile of recombinant protein. M: Protein marker; Lane 1: Protein resuspended in PBS after initial ammonium sulfate precipitation; Lane 2: Eluate from Diamond Butyl hydrophobic interaction chromatography (100% buffer); Lane 3: Eluate from Phenyl-30L hydrophobic interaction chromatography (80% buffer); Lane 4: Eluate from NanoGel-50Q ion-exchange chromatography (20% buffer); Lane 5: Eluate from NanoGel-50Q ion-exchange chromatography (30% buffer). (**c**) Transmission electron microscopy (TEM) image of recombinant SF protein at 100 nm scale. (**d**) Particle size distribution of recombinant SF protein analyzed by dynamic light scattering (DLS).

Recombinant plasmid pET-30a-SF was transformed into E.coli BL21(DE3) competent cells and induced to express by 0.25 mM IPTG at 25 °C. The bacterial lysates was analyzed by SDS-PAGE after ultrasound fragmentation. As illustrated in Figure 2a, distinct protein bands corresponding to ~30 kDa were observed in lanes 1–4, consistent with the theoretical molecular weight of the SF fusion protein. Notably, the majority of the recombinant protein partitioned into the soluble fraction (supernatant) upon centrifugation (12,000× *g*, 30 min), confirming efficient soluble expression.

After recombinant protein SF was purified by ammonium sulfate, in an Phenyl-30L column, NanoGel-50Q and Diamond Butyl, as shown in Figure 2b, the purity was the highest after ion-exchange NanoGel-50Q, so the protein was purified using this condition later in the experiment. Purified proteins were determined by Bicinchoninic acid assay to have a protein concentration of 1.25 mg/mL.

To verify the assembly effect of recombinant protein, the contour and granule morphology of recombinant protein were observed by transmission electron microscopy (TEM). As shown in Figure 2c, recombinant proteins can form many hollow, densely distributed, particles. Further analysis of their particle size by dynamic light scattering (DLS) showed that recombinant protein SFs could self-assemble to form spherical nanoparticles with a diameter of 16.2nm (Figure 2d).

### 3.3. Preparation and Characterization of SF-STG1 and SF-STG2 Nanoparticles

**Figure 3:**
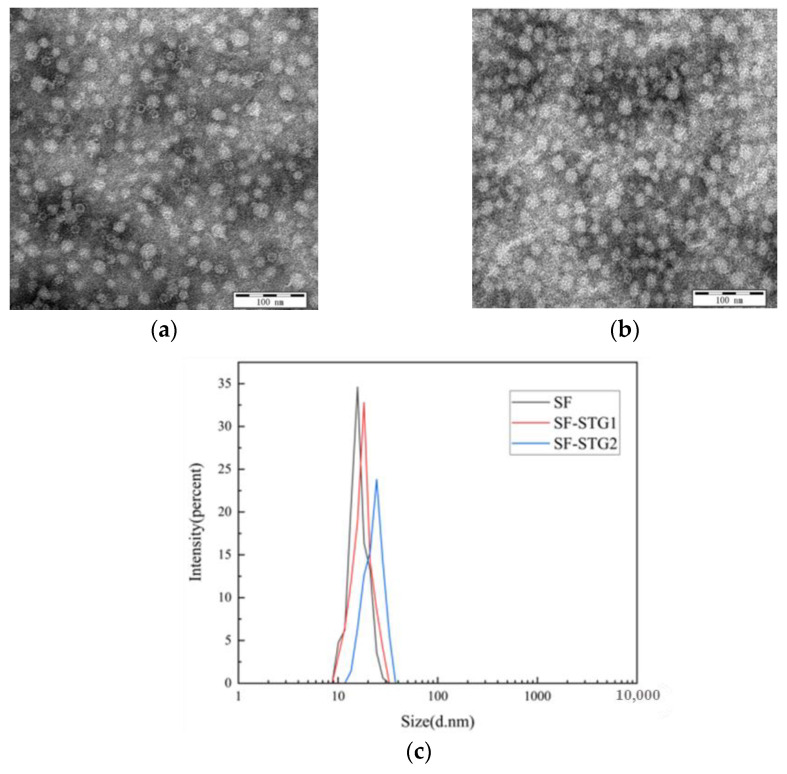
(**a**) Transmission electron microscopy (TEM) image of SF-STG1 nanoparticles. (**b**) Transmission electron microscopy (TEM) image of SF-STG2 nanoparticles. (**c**) Average diameter analysis of SF-STG1 and SF-STG2 nanoparticles.

The purified SF protein was conjugated with peptides STG1 and STG2 at a molar ratio of 1:8 overnight. Combined analysis using dynamic light scattering (DLS) and transmission electron microscopy (TEM) demonstrated preserved self-assembly capacity of the protein while achieving effective surface display of the conjugated peptides. As shown in Figure 3a,b, TEM imaging confirmed an increased particle size for SF-STG1 and SF-STG2. Consistent with this observation, DLS analysis (Figure 3c) demonstrated that SF-STG1 exhibited an average diameter of 17.8 nm, while SF-STG2 displayed an average diameter of 18.3 nm, compared to the native SF protein (16.2 nm). This size progression, consistent with steric effects from peptide conjugation, collectively confirms a surface display of STG1 and STG2 peptides on the SF protein scaffold.

### 3.4. Measurement of GnRH-Specific Antibody Levels in Serum

**Figure 4:**
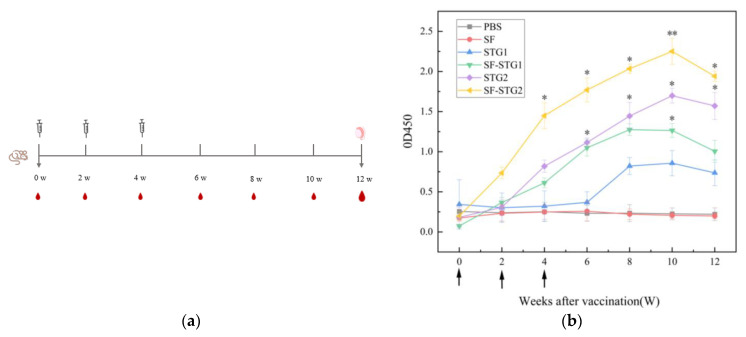
(**a**) Vaccination and sample collection schedule. (**b**) Measurement of serum GnRH-specific antibody levels in immunized and control groups. Arrows on the x-axis indicate vaccination time points at weeks 0, 2, and 4. (Data are presented as mean ± SD, n = 7 per group), * *p* < 0.05, ** *p* < 0.01, *** *p* < 0.001 (*t*-test).

GnRH-specific antibody levels in serum were detected every 2 weeks and analyzed using ELISA kits. As shown in Figure 4b, mice immunized with SF-STG1 and SF-STG2 nanoparticle vaccines exhibited GnRH-specific antibody production following the second immunization. The antibody titers progressively increased post-immunization, peaking at week 10 and remaining detectable until week 12. In contrast, serum samples from control group animals showed a complete absence of GnRH-specific immunoreactivity at all monitored time points.

### 3.5. Measurement of Serum Testosterone (T), Follicle-Stimulating Hormone (FSH), and Luteinizing Hormone (LH) Concentrations

**Figure 5:**
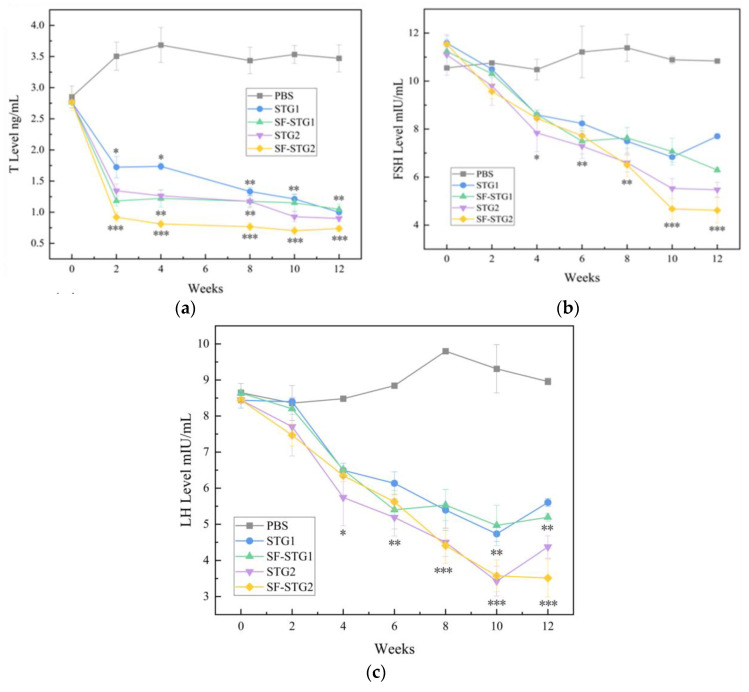
(**a**) Determination of serum T content (ng/mL). (**b**) Measurement of serum FSH levels (mIU/mL). (**c**) Determination of serum LH content (mIU/mL). (Data are presented as mean ± SD, n = 7 per group), * *p* < 0.05, ** *p* < 0.01, *** *p* < 0.001 (*t*-test).

Testosterone (T) concentrations in serum were detected every 2 weeks using ELISA kits. As shown in Figure 5a, T levels in all immunized groups gradually decreased post-immunization and remained significantly lower than those in the control group until 12 weeks after the first immunization (*p* < 0.1). Notably, the SF-STG2 group exhibited lower serum T concentrations compared to the SF-STG1 group.

Serum follicle-stimulating hormone (FSH) and luteinizing hormone (LH) concentrations were also assessed weekly. As demonstrated in Figure 5b,c, FSH and LH levels in immunized groups progressively declined starting at 2 weeks post-primary immunization, reaching minimal values by week 10, and remained suppressed until week 12. These levels were significantly lower than those in the blank control group (*p* < 0.05). Inter-group analysis revealed comparable efficacy between SF-STG1 and SF-STG2 formulations.

### 3.6. Testicular Weight and Histomorphological Determination

As shown in Table 1, terminal necropsy at week 12 revealed profound testicular atrophy in immunized cohorts. Compared to the vehicle control group (PBS group), vaccinated mice exhibited a significant 34.8% reduction in absolute testicular mass (*p* < 0.01). Testicular weights were 0.112 ± (0.01), 0.074 ± (0.01), and 0.077 ± (0.01) in the PBS group and the SF-STG1, SF-STG2 vaccine groups, with no significant difference between vaccine formulations.

**Figure 6:**
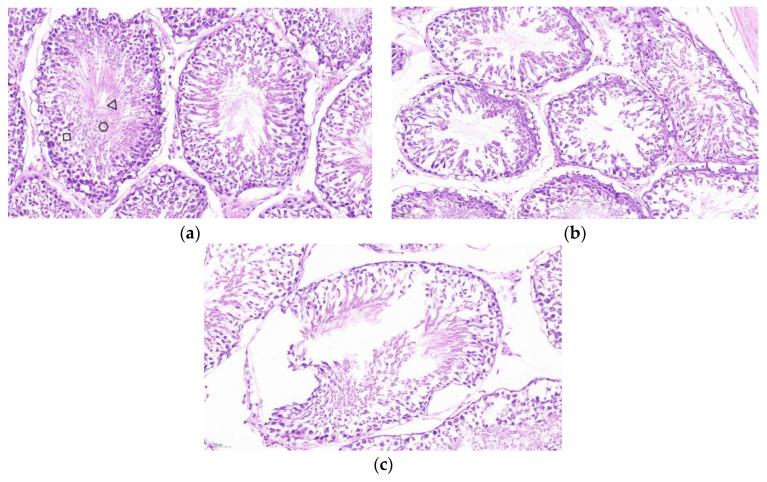
Histological analysis of testicular tissue sections with hematoxylin and eosin (HE) staining. (**a**) Testis from PBS control group mice. Boxes: spermatogonia, triangles: spermatocytes, circles: spermatozoa. (**b**) Testis from SF-STG1 immunized group mice. (**c**) Testis from SF-STG2 immunized group mice.

The testis was stained for HE sections. As shown in Figure 6a, the seminiferous tubules of the testis of PBS control mice developed normally, and the cells within the lumen developed and differentiated into distinct spermatogonia, primary spermatocytes, secondary spermatocytes, spermatids, and spermatozoa, with tightly arranged cells. As shown in Figure 6b,c, the cells in the lumen of the SF-STG1, SF-STG2 vaccine groups were sparse, with few cells at each developmental stage and almost no sperm.

### 3.7. Semen Quality Analysis

**Figure 7:**
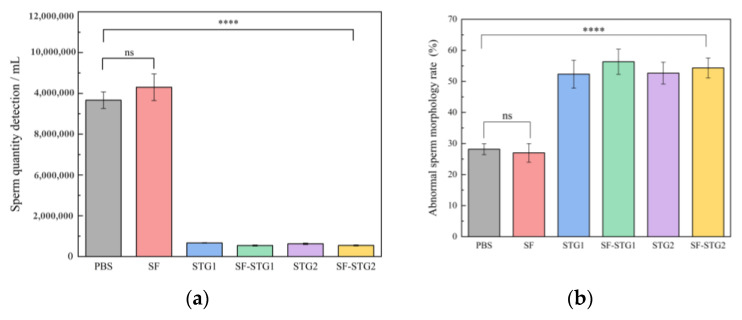
(**a**) Sperm count in the cauda epididymis of mice. (**b**) Analysis of sperm abnormality rate. (Data are presented as mean ± SD, n = 7 per group), * *p* < 0.05, ** *p* < 0.01, *** *p* < 0.001, **** *p* < 0.0001 (*t*-test).

The quality analysis of spermatozoa collected from the cauda epididymis revealed significant differences between immunized groups and control groups. As illustrated in Figure 7a, the mean sperm count in all immunized groups (2.0 ± 0.6 × 10^5^) was markedly lower than those in the PBS control group (7.7 ± 0.7 × 10^6^) and SF control group (8.3 ± 1.2 × 10^6^) (*p* < 0.001). Figure 7b demonstrates that the average sperm abnormality rate in immunized groups reached 61.7% ± 1.14%, which was higher than the 27.6% ± 2.4% observed in the control group (*p* < 0.001). These collective findings indicate that sperm quality in immunized groups was substantially inferior to that of the control groups.

### 3.8. Determination of GnRH-Specific Antibody Levels in Serum After Coupling with ISA206 Adjuvant

**Figure 8:**
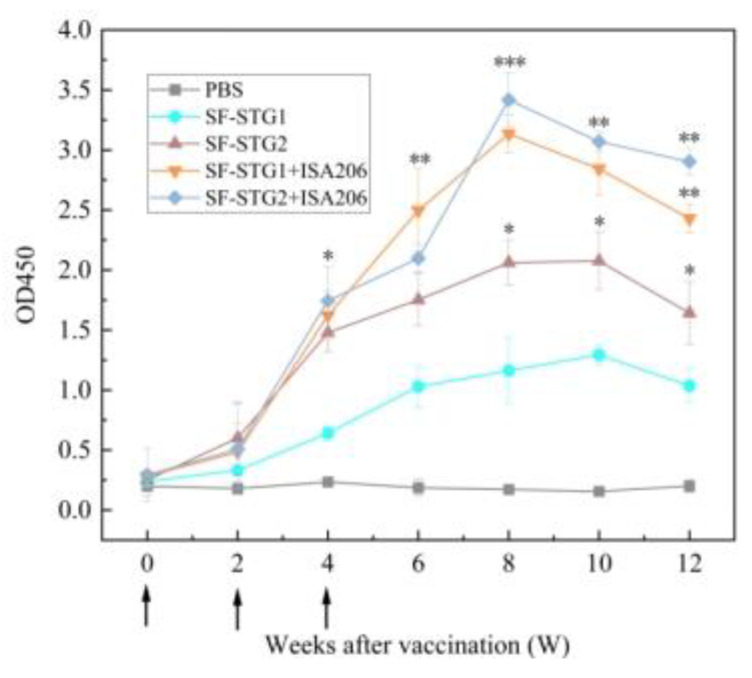
Measurement of serum GnRH-specific antibody levels in immunized and control groups. Arrows on the x-axis indicate vaccination time points at weeks 0, 2, and 4. (Data are presented as mean ± SD, n = 7 per group), * *p* < 0.05, ** *p* < 0.01, *** *p* < 0.001 (*t*-test).

The vaccination and sample collection schedule both follow what is described in Figure 4a, with only the administered vaccine formulations differing. The immune-enhancing effect of adjuvant formulations was evaluated by monitoring anti-GnRH IgG titers via enzyme-linked immunosorbent assay (ELISA). As depicted in Figure 8, both SF-STG1+ISA 206 and SF-STG2+ISA 206 nanovaccines exhibited GnRH-specific antibody production after the second immunization. Specific antibody levels gradually increased post-immunization, peaking at week 8, with the highest OD450 values of 3.2 for the SF-STG1+ISA 206 group and 3.4 for the SF-STG2+ISA 206 group. This immune response persisted until week 12 (*p* < 0.05). No anti-GnRH antibodies were detected in the control groups.

### 3.9. Determination of Serum Concentrations of T, FSH, and LH After Coupling with ISA206 Adjuvant

**Figure 9:**
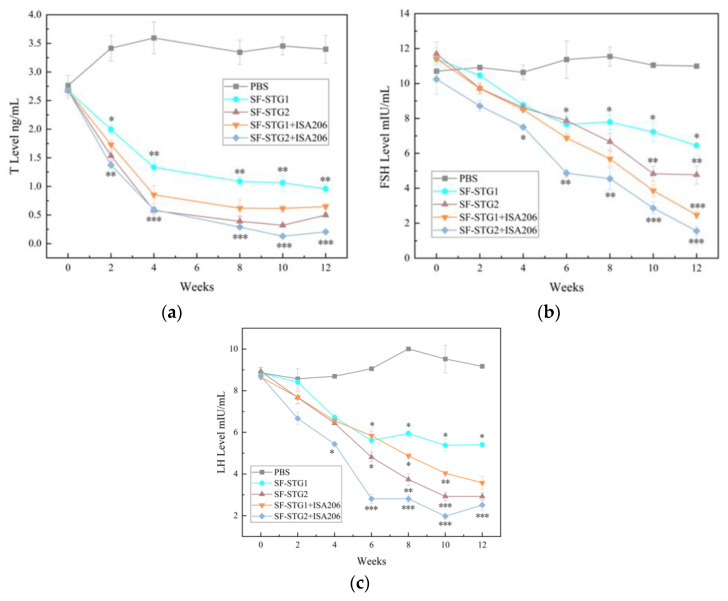
(**a**) Measurement of serum testosterone (T) levels. (**b**) Measurement of serum follicle-stimulating hormone (FSH). (**c**) Measurement of serum luteinizing hormone (LH) levels. (Data are presented as mean ± SD, n = 7 per group), * *p* < 0.05, ** *p* < 0.01, *** *p* < 0.001 (*t*-test).

Testosterone concentrations in serum after mixing with ISA206 adjuvant were detected every 2 weeks by ELISA kits. As shown in Figure 9a, T levels in each immunization group showed a downward trend after immunization and remained significantly lower than the concentration level in the control group. Notably, testosterone concentrations decreased from week 2 to less than 1 ng/mL in both the SF-STG1+ISA 206 and SF-STG2+ISA 206 groups with adjuvant addition and continued to be maintained until week 12 after the first immunization (*p* < 0.1).

The serum concentrations of FSH and LH were tested every 2 weeks by ELISA kits. As shown in Figure 9b,c, serum FSH and LH concentrations began to decline gradually 2 weeks after the first immunization, with the SF-STG1+ISA 206 and SF-STG2+ISA 206 nanoparticles vaccine groups reaching their lowest concentrations at the 10th week after immunization, significantly lower than the blank control group (*p* < 0.05).

### 3.10. Determination of Testicular Weight and Histomorphology After Coupling with ISA206 Adjuvant Terminal Necropsy

As shown in Table 2, terminal necropsy at week 12 revealed profound testicular atrophy in immunized cohorts. The testicular weight, length, and width in the immunized groups were significantly smaller than those in the control group. As shown in Table 1, the testicular weights of the PBS group, SF-STG1+ISA206, and SF-STG2+ISA206 vaccine groups were 0.109 g ± 0.01, 0.059 g ± 0.02, and 0.043 g ± 0.01, respectively. Compared to the control group, the testicular length and width in the vaccinated groups were significantly reduced.

**Figure 10:**
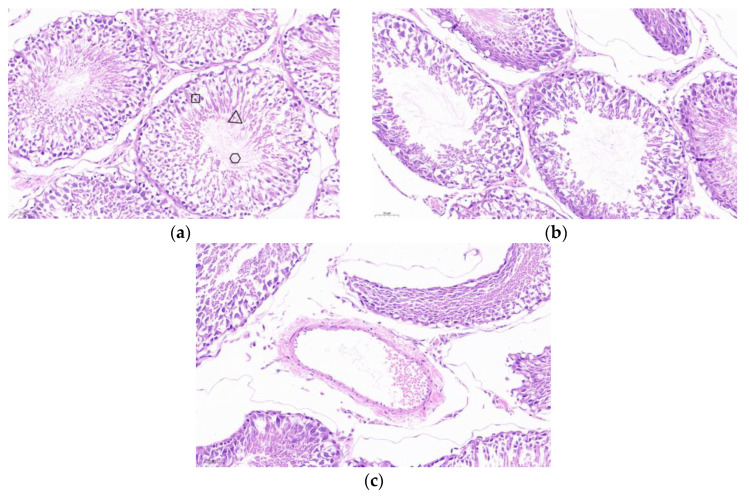
Testicular tissue sections and HE staining (**a**) PBS control mouse testis, box: spermatogonia, trigon: spermatids, circle: spermatozoa. (**b**) The testis of mice in the SF-STG1+ISA206 immune group. (**c**) The testis of mice in the SF-STG2+ISA206 immune group.

Hematoxylin-eosin (HE) staining was performed on testicular tissues. As illustrated in Figure 10a, the seminiferous tubules of PBS control mice exhibited normal development, with intact morphology of spermatogonia, spermatocytes, spermatids, and spermatozoa, as well as tightly arranged cells. In contrast, as shown in Figure 10b,c, the SF-STG1+ISA206 and SF-STG2+ISA206 vaccine groups displayed interstitial loosening, the vacuolization of seminiferous tubules, the segregation and depletion of spermatogenic cells at various levels within the lumen, and an almost complete absence of spermatozoa.

### 3.11. Analysis of Sperm Quality After Coupling with the ISA206 Adjuvant

**Figure 11:**
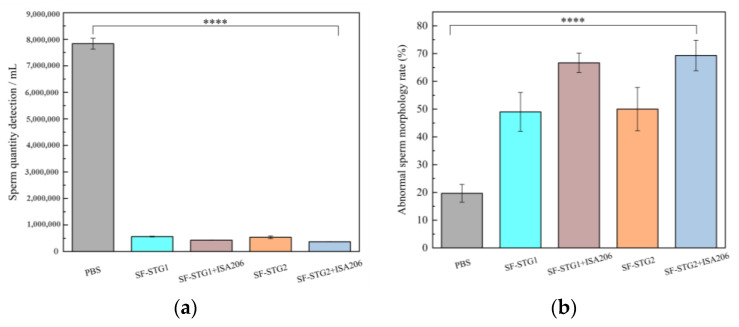
(**a**) Number of sperm counts in the mouse epididymis. (**b**) Analysis of sperm deformity rate. (Data are presented as mean ± SD, n = 7 per group), * *p* < 0.05, ** *p* < 0.01, *** *p* < 0.001, **** *p* < 0.0001 (*t*-test).

Quantitative spermatological assessment was conducted on epididymal spermatozoa harvested at the 12-week terminal endpoint. As shown in Figure 11a,b, the mean sperm count (4.7 ± 0.7 × 10^5^) was significantly reduced in all immunization groups compared with the PBS control group (7.8 ± 0.6 × 10^6^). Meanwhile, the mean sperm deformity rate in the immune group (58.7% ± 1.21%) was significantly higher than that in the control group (19.6% ± 3.1%, *p* < 0.001). These results show that the sperm count and morphology of the immune group were significantly impaired compared with the control group, indicating that there was a significant damage to sperm quality.

## 4. Discussion

Immune castration is a new animal-friendly, painless alternative castration technique, and GnRH has been successfully used in animal fertility control as an immune castration vaccine. The two major isomers of GnRH are known as GnRH I and GnRH II [25], and most studies on immune castration have targeted GnRH I [26,27,28], but studies have also shown that immunization of mice with recombinant GnRH I+II protein complexes produces higher antibody titers than recombinant GnRH I protein and recombinant GnRH II protein alone [29]. Based on this, two polypeptides were synthesized in this study: the polypeptide STG1 containing only GnRH I and the polypeptide STG2 containing GnRH I+II. Satish K. Gupta et al. showed [30] that the GnRH immunized female beagle dogs with appropriate mixed T cell epitope recombinant proteins, and the immune group produced high GnRH antibody titers, so we fused the T epitope at STG1. These two polypeptides constructed Spy Catcher-Spy Tag-based ferritin nano vaccines by coupling reactions, respectively, and both coupling proteins were able to form nanoparticles by TEM and DLS. The results of the mouse experiments showed that the SF-STG1 and SF-STG2 immune groups produced higher levels of specific GnRH antibodies compared with the polypeptides STG1 and STG2 alone, but the highest levels were in the SF-STG2 group. It is illustrated that the application of the SC-ST system with ferritin as a carrier effectively improves the immunogenicity of GnRH and that a synergistic effect of GnRH I-GnRH II is present.

LH directly stimulates testosterone secretion from mesenchymal cells, and FSH enhances the action of testosterone through supporting cells, and both synergize to maintain testicular function [31,32,33]. In this study, mice in the SF-STG1 and SF-STG2 immunized groups had significantly decreased concentrations of FSH and LH, and testicular weights and volumes of mice in the immunized groups were significantly lower than those in the control group. It suggests that our vaccine reduces the concentration of the downstream hormone testosterone and induces irreversible testicular atrophy by maintaining high levels of GnRH antibodies. Testicular development and function are dependent on testosterone secretion [6,34,35,36]. Microscopic observation of sections of mice testis showed that the testicular seminiferous tubules of control mice developed normally, and the spermatogonia in the lumen could develop and differentiate normally; there were almost no mature spermatozoa in the immunized group. Quality analysis of spermatozoa in the epididymis of mice in the 12th week showed that the average sperm count of each immunized group was significantly lower than that of the control group, and the rate of sperm malformation was high. These findings reflect the fact that reduced GnRH concentrations result in decreased fertility and defective testicular cell adhesion.

Several studies have demonstrated that the Spy Catcher-Spy Tag vector delivery system enables the coupled expression of antigens with nanocarriers and triggers high specific antibody titers and high neutralizing antibody levels [22,37,38,39]. Ma et al. [40] conjugated RBD and HR antigens in SARS-CoV-2 spike protein with Ferritin by using the Spy Catcher-Spy Tag system. This nanoparticle vaccine elicited a more effective neutralizing antibody response and a stronger T-cell immune response than the monomer vaccine. In this study, a carrier system based on Spy Tag/Spy Catcher and ferritin was applied for the first time to a castration vaccine, which successfully stimulated the production of specific GnRH IgG antibodies, decreased serum concentrations of testosterone, FSH, and LH, induced testicular shrinkage, and lowered sperm quality in mice. These results suggest that the application of the SC-ST system on the basis of the ferritin vector can enhance the immunogenicity of the GnRH monomer and strengthen the denervation effect.

The commercially available GnRH vaccine formulation GonaCon™ has been developed for application across a range of mammalian species [41]. It comprises GnRH conjugated to a carrier protein as the antigen, alongside purified Mycobacterium avium serving as the adjuvant. This adjuvant exhibits efficacy exclusively upon intramuscular administration and carries the potential for adverse effects [42]; for instance, GonaCon injections in canines have been shown to induce swelling at the injection site and muscle atrophy [43]. In contrast, MONTANIDE ISA 206 VG adjuvant—primarily utilized in veterinary vaccine formulations—functions by forming a stable oil-in-water emulsion structure. This structural property extends the retention duration of antigens at the injection site and enhances their uptake and processing by antigen-presenting cells (APCs).

Given these properties, we further immunized mice with a mixture of the nanoparticle vaccine and MONTANIDE ISA 206 VG adjuvant. The adjuvant-containing immunized groups showed superior performance compared to the non-adjuvant groups across multiple assays, including specific antibody levels and concentrations of testosterone, FSH, and LH. Serum analysis of GnRH antibody titers revealed that all groups exhibited a significant increase in antibody levels by 2 weeks after the initial immunization. Notably, the adjuvant-supplemented groups had significantly higher antibody levels than the non-adjuvant counterparts, with the SF-STG2+ISA 206 group showing the highest levels overall. Testosterone concentration assays revealed that testosterone levels in the immunized groups began to decline gradually from the second week post the first immunization, remaining significantly lower than those in the control group throughout the observation period. Notably, in the adjuvant-containing SF-STG1+ISA 206 and SF-STG2+ISA 206 groups, testosterone concentrations dropped below 1 ng/mL as early as the 2nd week and stayed beneath this threshold through the 10th week. Additionally, analyses of serum follicle-stimulating hormone (FSH) and luteinizing hormone (LH) concentrations demonstrated that the adjuvant-containing immunized groups exhibited significantly lower levels of these hormones compared to the control group. The above results indicate that based on the ferritin vector, the application of the SC-ST system connected and mixed with adjuvant can effectively reduce serum testosterone, FSH, and LH concentrations, leading to fertility suppression and a de-escalation effect.

In both SF-STG1 and SF-STG2 groups, after the first 10 weeks of immunization, the level of specific GnRH antibody in serum began to decrease, and the testosterone concentration began to show an increasing trend. This phenomenon may be attributed to the fact that low antibody titers can inhibit testosterone production during the immature development of the reproductive system in mice, but with the gradual development of the complete reproductive system and the termination of the immunization program, the immune de-escalation occurred as a reversible phenomenon. This has also been reported before [44,45,46,47].

## 5. Conclusions

Taken together, our study identifies the Spy Catcher-Spy Tag-ferritin platform as a general strategy for GnRH vaccine development, achieving long-term inhibitory effects of the hormone and a significant decrease in sperm quality through GnRH I-GnRH II synergy, mixed with ISA 206 adjuvant, with superior efficacy to conventional monovalent preparations. Future explorations of the immune validity period of this vaccine are also needed, along with the composite use of adjuvants or the use of other types of adjuvants.

## Figures and Tables

**Table 1:** Comparison of testicular weight, length, and width among groups (mean ± SD, n = 7). (*t*-test). (All *p*-values in the table are only for comparisons between the SF-STG2 group and the PBS group).

N = 7	Testis Weight (g ± SD)	Testis Length (cm ± SD)	Testis Width (cm ± SD)
PBS	0.112 ± (0.01)	0.76 ± (0.05)	0.54 ± (0.02)
SF	0.117 ± (0.01)	0.75 ± (0.01)	0.54 ± (0.01)
STG1	0.087 ± (0.01)	0.59 ± (0.05)	0.46 ± (0.05)
SF-STG1	0.074 ± (0.01)	0.58 ± (0.02)	0.41 ± (0.03)
STG2	0.084 ± (0.01)	0.59 ± (0.02)	0.45 ± (0.02)
SF-STG2	0.077 ± (0.01)	0.59 ± (0.02)	0.41 ± (0.03)
*p*-value	*p* < 0.05	*p* < 0.001	*p* < 0.001

**Table 2:** Comparison of testicular weight, length, width, and volume among groups (mean ± SD, n = 7). (*t*-test). (All *p*-values in the table are only for comparisons between the SF-STG2+ISA206 group and the PBS group).

N = 7	Testis Weight (g ± SD)	Testis Length (cm ± SD)	Testis Width (cm ± SD)
PBS	0.109 ± (0.01)	00.74 ± (0.03)	0.52 ± (0.02)
SF-STG1	0.091 ± (0.01)	0.75 ± (0.01)	0.42 ± (0.01)
SF-STG1+ISA206	0.059 ± (0.02)	0.62 ± (0.05)	0.41 ± (0.05)
SF-STG2	0.074 ± (0.01)	0.58 ± (0.02)	0.39 ± (0.03)
SF-STG2+ISA206	0.043 ± (0.01)	0.53 ± (0.02)	0.37 ± (0.02)
*p*-value	*p* < 0.05	*p* < 0.001	*p* < 0.001

## Data Availability

The data presented in this study are contained within the article.
